# Improving the use of research evidence in guideline development: 4. Managing conflicts of interests

**DOI:** 10.1186/1478-4505-4-16

**Published:** 2006-12-01

**Authors:** Elizabeth A Boyd, Lisa A Bero

**Affiliations:** 1 Department of Clinical Pharmacy, University of California, San Francisco, 3333 California Street, Suite 420, San Francisco, CA 94143-0613, USA; 2 Department of Clinical Pharmacy and the Institute for Health Policy Studies, University of California, San Francisco, Suite 420, Box 0613, 3333 California Street, San Francisco, CA 94118, USA

## Abstract

**Background:**

The World Health Organization (WHO), like many other organisations around the world, has recognised the need to use more rigorous processes to ensure that health care recommendations are informed by the best available research evidence. This is the fourth of a series of 16 reviews that have been prepared as background for advice from the WHO Advisory Committee on Health Research to WHO on how to achieve this.

**Objectives:**

We reviewed the literature on conflicts of interest to answer the following questions:

1. What is the best way to obtain complete and accurate disclosures on financial ties and other competing interests?

2. How to determine when a disclosed financial tie or other competing interest constitutes a conflict of interest?

3. When a conflict of interest is identified, how should the conflict be managed?

4. How could conflict of interest policies be enforced?

**Methods:**

We searched PubMed, the Cochrane Methodology Register and selectively searched for the published policies of several organizations, We did not conduct systematic reviews ourselves. Our conclusions are based on the available evidence, consideration of what WHO and other organisations are doing and logical arguments.

**Key questions and answers:**

What is the best way to obtain complete and accurate disclosures on financial ties and other competing interests?

• Although there is little empirical evidence to guide the development of disclosure forms, minimal or open-ended formats are likely to be uninformative. We recommend the development of specific, detailed, structured forms that solicit as much information as possible about the nature and extent of the competing interests.

How to determine when a disclosed financial tie or other competing interest constitutes a conflict of interest?

• There is no empirical evidence to suggest that explicit criteria are preferable to ad hoc committee decisions when deciding if a disclosed financial tie is a conflict of interest. However, explicit criteria may make decision-making easier.

When a conflict of interest is identified, how should the conflict be managed?

• Descriptive studies suggest that appropriate management strategies are best determined on a case-by-case basis. Thus, WHO should use a wide range of management strategies to address disclosed conflicts of interest, with public disclosure of conflicts associated with each meeting as a minimum and recusal of conflicted individuals as the other extreme.

How could conflict of interest policies be enforced?

• Although there are no empirical studies of the enforcement of conflict if interest policies, descriptive studies of other organizations and institutions suggest that WHO convene a standing committee to review all financial disclosure statements prior to the commencement of committee meetings/hearings and to make management recommendations when necessary. A standard policy requiring all financial ties to be made public (i.e., recorded into the meeting minutes) should reduce the number of problematic cases. In instances where the conflicts seem intractable, a recommendation of recusal may be necessary to protect the greater interests of WHO and its constituents.

## Background

The World Health Organization (WHO), like many other organisations around the world, has recognised the need to use more rigorous processes to ensure that health care recommendations are informed by the best available research evidence. This is the fourth of a series of 16 reviews that have been prepared as background for advice from the WHO Advisory Committee on Health Research to WHO on how to achieve this.

A conflict of interest exists when an individual's secondary interests (e.g. personal financial) interfere with or influence judgments regarding the individual's primary interests (e.g. patient welfare, education, research integrity). There is evidence demonstrating the association of financial ties with a breakdown in research integrity. Recent studies and reviews have found that industry funding for research is associated with favourable outcomes for the sponsor [1-5]. Financial ties of investigators with their sponsors (stock ownership, consulting income, etc.) are also associated with favourable research outcomes for the sponsor [5]. This scholarly evidence has been accentuated by lay media stories documenting how financial conflicts of interest have led to biased and even dangerous research (e.g., [6,7]). Biased research may be intentional or unintentional [8] and may result from damaged objectivity at multiple stages in the research process, including conceptualization of the question, design or conduct of the research, interpretation of the results, and publication (or not) of the research [9,10]. Regardless of its source, the bias associated with financial and other conflicts of interest may damage both the public's and other researcher's trust in science [11]. The type of conflict most likely to affect the public's trust is a financial conflict where the scientist tends to gain financially from a particular research outcome [11-16], although other competing interests, such as professional advancement, are important. Conflict of interest policies are designed to protect the integrity of research and decision-making processes through disclosure and transparency.

The following report relies heavily on published research related to conflicts of interest in the context of U.S. academic research and U.S. and U.K. biomedical journals because there is little empirical research from other areas.

In this paper we address the following questions:

• What is the best way to obtain complete and accurate disclosures on financial ties and other competing interests?

• How to determine when a disclosed financial tie or other competing interest constitutes a conflict of interest?

• When a conflict of interest is identified, how should the conflict be managed?

• How could conflict of interest policies be enforced?

Related questions about group composition, consultation and group processes are addressed in another paper in this series [17,18].

## What WHO is doing now

Expert Advisory Panel members are currently required to disclose "all circumstances that could give rise to a potential conflict of interest as a result of their membership on an expert committee." [19]

According to the WHO Declaration of Interests for WHO Experts, a conflict of interest occurs when "the expert or his/her partner (a spouse or other person with whom s/he has a similar close personal relationship), or the administrative unit with which the expert has an employment relationships, has a financial or other interest that could unduly influence the expert's position with respect to the subject matter being considered." An apparent conflict of interest exists when the existence of an interest could result in the expert's objectivity being questioned by others, and a "potential conflict of interest exists with an interest which any reasonable person could be uncertain whether or not should be reported" [20].

The Declaration identifies 5 types of financial and other interests that must be disclosed by all experts, including proprietary interests and patents, shares or bonds in a related commercial entity, employment or consultancies, paid work or research, and grants or fellowships from a commercial entity that has an interest in the subject-matter or work of the committee. [20]

We are not aware of specific WHO documents providing guidance on how to avoid or manage conflicts of interest, and we know of no processes required to ensure that the committees discuss potential conflicts of interest on a case-by-case basis and handle them appropriately. There may be some variability in how departments collect and manage the disclosed information.

In October 2005, the WHO Office of Legal Counsel recommended a set of proposed revisions to the existing conflict of interest procedures that are similar to the recommendations in our report. These revisions would clarify the definition of a conflict of interest, include recommendations for avoiding situations that might result in conflicts of interest, and expand the relationships and affiliations that must be disclosed. The draft guidelines also recommend that a determination be made as to whether the expert's declared interest is insignificant, clearly significant, or potentially significant (para. 26). Suggestions for making this determination include weighing the nature and extent of the interest, the context of the work, and the importance of the expert's contribution (para. 29). The draft Guidelines also suggest three options for managing a conflict: 1) continue with public disclosure of the interest; 2) limit the expert's involvement; or 3) exclude the expert from the meeting or work altogether (para. 30)[21]

The draft guidelines also include a requirement that WHO experts disclose ties to the tobacco industry. This recommendation is in response to a 2000 commissioned report investigating the influence of the tobacco industry on WHO's global tobacco control policies. That report recommended that WHO formally vet prospective experts, consultants, and advisers for possible conflicts of interest related to the tobacco industry and that staff should be barred from having links with the tobacco industry [22]. In 2003, WHO's hypertension guidelines were revised in response to criticism about possible conflicts of interest among expert members [23].

## What other organizations are doing

Many organizations recognize the importance of protecting against actual and potential conflicts of interest and require special employees, advisory committee members, and participants to disclose their financial ties to the organization. For example, the US Food and Drug Administration (FDA), the Cochrane Collaboration, the UK National Institute for Health and Clinical Excellence (NICE), and the US National Academies of Science all require advisory committee members and other special participants to disclose financial relationships, including research sponsorship, equity ownership, consulting fees, honoraria, related to the work or topic of the committee. These organizations use a structured disclosure form to solicit information; they employ different standards for determining conflicts of interest and for managing them (see below).

## Methods

The methods used to prepare this review are described in the introduction to this series [24] Briefly, the key questions addressed in this paper were vetted amongst the authors and the ACHR Subcommittee on the Use of Research Evidence (SURE). We searched PubMed and the Cochrane Methodology Register [25] for existing systematic reviews and relevant methodological research that address these questions. We did not conduct systematic reviews ourselves. The answers to the questions are our conclusions based on the available evidence, consideration of what WHO and other organisations are doing, and logical arguments.

For this review, we searched PubMed for original qualitative and quantitative research using the terms "conflicts of interest" and "disclosure" and the Cochrane Methodology Register using "conflict of interest". We searched the reference lists of all relevant publications, consulted references from the Council of Medical Editors meeting on disclosure (Sept 2004) and selectively searched for the published policies of several organizations, including the Cochrane Collaboration, NICE, FDA, and National Academies of Science [26-30].

## Findings

Our database searches did not yield any systematic reviews of conflict of interest or financial disclosure policies. We found several systematic reviews of literature examining the association between commercial sponsorship and outcomes favorable to the sponsor and the financial ties of investigators and favorable outcomes. We also found a number of empirical studies of particular aspects of industry involvement in science and medicine, case studies and commentaries.

### What is the best way to obtain complete and accurate disclosures on financial ties and other competing interests?

We were unable to identify any randomized, controlled trials or other rigorous studies evaluating different methods for obtaining conflict of interest disclosures. Biomedical journals gather financial interest statements from authors of submitted manuscripts in three ways: 1) minimal requests about authors' professional and financial affiliations that may be perceived to have biased the presentation of results; 2) detailed instructions that request authors to describe all involvements with organizations or entities with direct financial interest in the subject matter of the study; and 3) detailed, structured checklists that require authors to declare specific interests [31]. Krimsky and others are critical of the utility of minimal and open-ended requests [31]. Bero et al. caution that simple disclosure requests may not reveal the nature and extent to which commercial interests exert influence over the scientific process [32].

The Cochrane Collaboration Steering Group members, the Food and Drug Administration advisory committee members, NICE, and the National Academies use structured disclosure forms and request information on a range of financial ties, including research funding, paid consultancies, honoraria, equity holdings, gifts, patents, and royalties. The Cochrane Collaboration also requests information on positions of management in a related entity, including service as a director, officer, partner, trustee or employee, and information on outstanding loans from the entity. The National Academies of Science request disclosure of any position that would give the individual access to confidential information, including patient records, classified and proprietary information. NICE requests information regarding an individual's private practice that could be affected by the outcome or discussion of a particular matter or product.

There is considerable variation along other dimensions of disclosure as well. These include:

• When disclosures should be made: Upon appointment to the committee? Prior to the start of committee work? Under each agenda item? At the start of each committee meeting?

• What level of financial interest should be disclosed: Any amount (>US$0)? Over US$250/per year in annual income? Over US$10,000 in equity holdings? Exact amounts or ranges (i.e., US$1000–$5000)?

• What period of time should be covered by the disclosure: The current calendar year? Past 12 months? Past 5 years? Past 5 years and future 2 years?

• Who should the disclosure cover: Individual only? Individual's spouse and children? Individual's institution?

### How to determine when a disclosed financial tie or other competing interest constitutes a conflict of interest?

Few organizations or institutions provide explicit guidelines for determining when a particular financial relationship constitutes a conflict of interest. The Association of American Medical Colleges prohibits financial relationships between principal investigators and commercial sponsors of clinical trials, but uses a "rebuttable presumption" clause to allow the prohibition to be waived when the benefits of the research outweigh the risks of the conflict of interest [33]. The US National Institutes of Health and National Science Foundation establish financial thresholds for disclosure – $10,000 in annual income or 5% equity ownership in a commercial entity related to the scientific work [34].

The US FDA does not prohibit financial relationships among its Advisory Committee members and regularly issues waivers for disclosed conflicts of interest when 1) "the disqualifying financial interest is not so substantial that it is likely to affect the integrity of an employee's services to the government;" and 2) the "need for the employee's services outweighs the potential conflicts of interest" [29]. In making these determinations, the FDA evaluates "the type of interest creating the disqualification; the identity of the person whose financial interest is at issue; the dollar value of the disqualifying financial interest including its value in relationship to the individual's overall assets; the nature and importance of the individual's role in the matter, including the extent to which the employee is called upon to exercise discretion; the sensitivity of the matter; and the need for the employee's services in the particular matter" [29].

These criteria are in line with the criteria used by University of California conflict of interest committees. In the only empirical study to date of how conflict of interest committees define and manage disclosed financial relationships of faculty investigators [35], found that committees typically examined the nature of the proposed scientific work (basic or applied), the overlap between paid activities and the research topic, the length and dollar amount of the relationship between the investigator and the commercial entity, and the degree to which the investigator could be seen as independent of the company's interests.

The overall lack of explicit criteria for determining which relationships constitute conflicts of interest reflect a common perception that these decisions should be made on an ad hoc basis and that the organization must always balance its own needs for the particular expertise of the individual with the needs of the public (in terms of advancing scientific discovery as well as trust in the scientific process). Little is known, however, about the needs and understandings of the public in this regard. The few studies we have identified to date provide evidence of both favorable and unfavorable reactions of the public [36,37]. Professionals with industry ties are more supportive of financial ties than those without industry ties. Investigators recognize general risks of conflicts of interest, but not for themselves. Investigators tend to support disclosure of financial ties, although there is evidence that disclosure leads to more critical review of research findings [38]. Schroter and colleagues showed that the overall importance, relevance, validity, and believability of studies disclosing competing interests were rated lower by readers than those without competing interests. [39]

### When a conflict of interest is identified, how should the conflict be managed?

The only empirical studies of management decisions in conflicts of interest detail a number of management strategies that are commonly used by university conflict of interest committees [35,40]. These possible management strategies include: disclosure of the financial tie(s) in publications and public presentations; reducing equity holdings to below 5%; altering consulting agreements to ensure separation between consulting and research work; eliminating the financial tie; appointing oversight committees to review the scientific process and resulting research; and recusal. Government and professional society guidelines recommend that institutions "manage" the financial conflicts of interest of their researchers. Disclosure of financial ties in all publications and presentations is the most frequently used management strategy [40-43].

Scientific journals are also encouraging disclosure as a way of dealing with financial conflicts of interest [44], however, the adequacy of disclosures in scientific articles has been questioned [32,45]. Even when financial sponsorship is disclosed, few studies describe the role of the sponsor [46]. A study of the relationships between authors of clinical practice guidelines and the pharmaceutical industry found considerable interaction between guideline authors and the pharmaceutical industry [47]; another study found that clinical practice guidelines published in journals almost never published conflict of interest statements along with the guidelines [48].

The FDA in 2002 issued draft guidance amending their disclosure regulations related to Advisory Committee members. The draft guidance now requires that Advisory Committee members granted waivers of their conflicts of interest will have the nature and magnitude of their conflicts of interest disclosed and read into the public record at the start of the committee hearings. [28]. NICE, which is currently reviewing its policies on disclosure and conflicts of interests, recommends that "members should declare all interests at the beginning of all appraisals" and that those declarations of interests be kept in files available for public scrutiny or are recorded in the minutes of the meeting [27]. The Cochrane Collaboration publishes the declarations of interests of its Steering Group members [26].

Although disclosure of financial ties is becoming more accepted within the scientific and policy communities, there are widely varying opinions about the adequacy of disclosure as a management strategy for financial conflicts of interest. Some critics of disclosure feel that it is unnecessary and can taint the reputation of "good" researchers [49,50]. Others believe that "the key to avoiding conflict of interest is public disclosure" [51]. Studies that disclose industry sponsorship have a systematic bias towards outcomes that favor the sponsor [3,5,52,53], so, therefore, disclosure does not eliminate bias. Although disclosure does not eliminate the association of research funding with outcomes favorable to the sponsor, many argue that it can minimize perceived conflicts of interest.

Additional research is necessary to be able to evaluate different methods for defining conflicts of interest and to determine their relative impact on the decision-making capabilities of the organization.

### How should conflict of interest policies be enforced?

There is no empirical evidence evaluating the enforcement of conflict of interest policies. Most organizations and academic institutions convene a standing or ad hoc committee to review financial interest disclosures and, where deemed necessary, recommend management strategies. The US FDA vets all financial disclosure statements through a multi-stage process, beginning with initial review, followed by consultation with the individual and an FDA official, review by the FDA Ethics staff, and final approval by the appointing official. The FDA operates under federal regulations and thus has the power to enforce its decisions [28,29]. The Cochrane Collaboration directs unclear cases of financial disclosure for reviews to a "Funding Arbiter" who convenes a standing panel of four to give guidance [26].

## Further work

There is currently a lack of empirical evidence regarding the most effective ways to determine the existence of conflicts of interest, manage conflicts of interest, and enforce conflict of interest policies. Additional research is necessary to evaluate different methods for defining conflicts of interest and to determine their relative impact on the decision-making capabilities of the organization. WHO's proposed draft recommendations (October 7, 2005) represent a more rigourous evaluation of conflict of interest because it requires more complete disclosure, clearer standards for evaluating conflicts of interest, and explicit management strategies.

## Competing interests

The author(s) declare that they have no competing interests.

## Authors' contributions

EAB prepared the first draft and revisions of this paper. LAB contributed to drafting and revising it.
